# The Amsterdam Resting-State Questionnaire reveals multiple phenotypes of resting-state cognition

**DOI:** 10.3389/fnhum.2013.00446

**Published:** 2013-08-08

**Authors:** B. Alexander Diaz, Sophie Van Der Sluis, Sarah Moens, Jeroen S. Benjamins, Filippo Migliorati, Diederick Stoffers, Anouk Den Braber, Simon-Shlomo Poil, Richard Hardstone, Dennis Van't Ent, Dorret I. Boomsma, Eco De Geus, Huibert D. Mansvelder, Eus J. W. Van Someren, Klaus Linkenkaer-Hansen

**Affiliations:** ^1^Department of Integrative Neurophysiology, Center for Neurogenomics and Cognitive Research, VU University AmsterdamAmsterdam, Netherlands; ^2^Neuroscience Campus AmsterdamAmsterdam, Netherlands; ^3^Department of Functional Genomics, Center for Neurogenomics and Cognitive Research, VU University Amsterdam and VU Medical Center AmsterdamAmsterdam, Netherlands; ^4^Department of Sleep and Cognition, Netherlands Institute for NeuroscienceAmsterdam, Netherlands; ^5^Department of Biological Psychology, VU University AmsterdamAmsterdam, Netherlands; ^6^Department of Medical Psychology, VU Medical Center AmsterdamAmsterdam, Netherlands

**Keywords:** consciousness, EEG, introspection, mental health, mind wandering

## Abstract

Resting-state neuroimaging is a dominant paradigm for studying brain function in health and disease. It is attractive for clinical research because of its simplicity for patients, straightforward standardization, and sensitivity to brain disorders. Importantly, non-sensory experiences like mind wandering may arise from ongoing brain activity. However, little is known about the link between ongoing brain activity and cognition, as phenotypes of resting-state cognition—and tools to quantify them—have been lacking. To facilitate rapid and structured measurements of resting-state cognition we developed a 50-item self-report survey, the Amsterdam Resting-State Questionnaire (ARSQ). Based on ARSQ data from 813 participants assessed after 5 min eyes-closed rest in their home, we identified seven dimensions of resting-state cognition using factor analysis: Discontinuity of Mind, Theory of Mind, Self, Planning, Sleepiness, Comfort, and Somatic Awareness. Further, we showed that the structure of cognition was similar during resting-state fMRI and EEG, and that the test-retest correlations were remarkably high for all dimensions. To explore whether inter-individual variation of resting-state cognition is related to health status, we correlated ARSQ-derived factor scores with psychometric scales measuring depression, anxiety, and sleep quality. Mental health correlated positively with Comfort and negatively with Discontinuity of Mind. Finally, we show that sleepiness may partially explain a resting-state EEG profile previously associated with Alzheimer's disease. These findings indicate that the ARSQ readily provides information about cognitive phenotypes and that it is a promising tool for research on the neural correlates of resting-state cognition in health and disease.

## Introduction

The growing use of resting-state neuroimaging has greatly improved our knowledge of the spatiotemporal character of ongoing brain activity in health and disease. The observation of a highly active distributed set of brain regions during low-demanding tasks—referred to as the default mode network—has been particularly influential in the development of this field (Raichle et al., 2001). Many other resting-state networks (Damoiseaux et al., 2006; Smith et al., 2009) and their interactions (Fox and Raichle, 2007; Doucet et al., 2012) have since been described. Also the complex temporal dynamics of ongoing activity has received a great deal of interest (Linkenkaer-Hansen et al., 2001; He, 2011; Hardstone et al., 2012) with recent studies showing that that intrinsic neuronal dynamics during rest can predict individual variability in behavior (Palva et al., 2013; Smit et al., 2013). Testing whether individual variation in ongoing brain activity can also explain individual differences in non-sensory experiences would require tools to quantify thoughts and feelings based on introspection.

From a cognitive viewpoint, the resting state may be viewed as a model system for states in which attention drifts away from the task at hand towards inner mentation—also referred to as stimulus-independent thought (Antrobus, 1968; Teasdale et al., 1995), daydreaming, or mind wandering (Smallwood and Schooler, 2006). It has been proposed that this mentation could be linked to default mode network activity (Buckner et al., 2008) and, indeed, there is empirical support for this idea (Mason et al., 2007; Christoff et al., 2009; Hasenkamp et al., 2012). Still, even though mind wandering has been associated with attention, episodic memory retrieval, prospection, and theory of mind (Buckner and Carroll, 2007; Smallwood et al., 2013), remarkably little progress has been made in terms of developing “the resting state” as a multi-faceted cognitive construct with standardized tools to quantify it and, thereby, allowing to investigate its neuronal origin.

Several studies have used resting-state neuroimaging to identify pathophysiological states in brain disorders (Linkenkaer-Hansen et al., 2005; Stam et al., 2006; Greicius et al., 2007). It remains unknown, however, whether patients and healthy controls experienced the scanning session differently. If so, quantifying patient-control differences in cognitive states could help understand the functional significance of the observed physiological changes. However, systematic group differences in the elicited cognitive state could also lead to smaller differences in resting-state brain activity than would have been observed if the groups were balanced for their cognitive states. Furthermore, cognitive states that are secondary to the clinical condition, such as arousal or comfort (Ogedegbe et al., 2008), could bias the dynamic measures derived from functional neuroimaging data. In all of these situations, information about participants' cognitive state could potentially improve the interpretation or increase the sensitivity and specificity of neuroimaging biomarkers in clinical of pharmacological studies (Klumpers et al., 2012).

Here, we introduce the self-report Amsterdam Resting-State Questionnaire (ARSQ), consisting of 50 statements on thoughts and feelings that one may experience during rest. Based on a large ARSQ data set obtained using the online platform of the Netherlands Sleep Registry we developed a model of resting-state cognition using exploratory and confirmatory factor analysis (EFA and CFA). A primary motivation behind the conception of the ARSQ was to create an effective tool to be used in combination with neuroimaging. Therefore, we also tested the model of resting-state cognition on ARSQ data gathered from studies measuring resting-state functional Magnetic Resonance Imaging (fMRI) and electroencephalography (EEG). In addition, we studied the relations between the different dimensions of resting-state cognition and widely used measures of mental health, depression, anxiety and sleep quality. Our findings suggest that resting-state cognition is characterized by at least seven phenotypes, which we labeled Discontinuity of Mind, Theory of Mind, Self, Planning, Sleepiness, Comfort, and Somatic Awareness. Given the prominence of mind wandering in daily life (Killingsworth and Gilbert, 2010) and its putative impairment in the form of excessive worry and rumination in brain disorders (Mor and Winquist, 2002; Aldao et al., 2010), an efficient and validated tool that quantifies resting-state experience, such as the ARSQ, should be of interest to a wide range of clinical and cognitive scientists and, in particular, the resting-state neuroimaging community.

## Materials and methods

### Self-report amsterdam resting-state questionnaire (ARSQ)

Inspired by previous mind-wandering research (Buckner and Carroll, 2007), mindfulness scales (Brown and Ryan, 2003) and personality inventories (Costa and McRae, 1985), we initially formulated more than 100 Likert-type statements relating to thoughts and feelings that may be experienced during a resting state. All statements were scored on a five-point ordinal scale with the labels “Completely Disagree,” “Disagree,” “Neither Agree nor Disagree,” “Agree,” and “Completely Agree.” Small-sample pilot experiments helped identify statements that were ambiguous or too specific (e.g., items that were always rated “strongly disagree”) and participants were asked to suggest statements they felt were lacking. Eventually, a subset of 45 statements was selected for the ARSQ (Table 1 and an additional 5 items were selected to assess response validity, bringing the total length of the ARSQ to 50 statements. A key aim during the questionnaire/scale development was to minimize the impact of the fading memory of the resting-state experience by keeping the questionnaire short: the ARSQ is usually completed in less than 4 min even when lying in an fMRI scanner (226 ± 39 s SD). The item order of the ARSQ was randomized in the fMRI and EEG groups.

**Table 1:** **Endorsement rate of ARSQ items (*n* = 1367, complete data set)**.

**ARSQ Item #**	−−	−	+/−	+	++
“I thought about my feelings.”	24	27	21	24	5
“I felt restless.”	32	24	20	17	7
“I felt tired.”	28	25	20	20	7
“I felt sleepy.”	24	30	18	21	7
“I felt comfortable.”	3	12	20	51	15
“I felt relaxed.”	4	15	20	46	16
“I felt happy.”	6	15	42	29	7
“I felt ill.” (R)	72	18	7	3	1
“I enjoyed the session.”	14	22	36	22	6
“I had negative feelings.” (R)	45	31	14	9	1
“I felt bored.”	26	30	21	19	5
“I felt nothing.”	28	36	26	9	1
“I felt the same throughout the session.”	5	19	16	47	12
“I thought about my health.”	39	30	12	17	2
“I thought about my work/study.”	36	25	10	24	5
“I thought about my behavior.”	34	30	13	20	3
“I had thoughts that I would not readily share with others.”	53	32	7	5	2
“I had busy thoughts.”	28	30	18	17	7
“I had similar thoughts throughout the session.”	7	19	22	41	10
“I thought about others.”	17	25	18	34	5
“I thought about myself.”	9	12	19	51	8
“I thought about pleasant things.”	11	21	33	31	5
“I had my thoughts under control.”(R)	7	19	26	39	9
“I thought about solving problems.”	25	30	18	24	3
“I thought about the aim of the experiment.”	29	26	16	25	5
“I had difficulty staying awake.”	47	34	10	7	3
“I had rapidly switching thoughts.”	14	24	21	29	11
“I had superficial thoughts.”	8	24	25	38	4
“I thought about the past.”	35	36	12	14	3
“I thought about the present.”	10	10	18	51	11
“I thought about the future.”	22	26	19	29	5
“I had deep thoughts.”	27	39	21	11	2
“I thought about nothing.”	41	29	16	11	4
“I had difficulty holding on to my thoughts.”	18	30	24	24	4
“I thought about people I like.”	26	24	19	25	6
“I thought in images.”	22	20	16	32	10
“I thought in words.”	14	21	18	36	11
“I thought about things I need to do.”	19	22	16	32	11
“I was conscious of my body.”	8	13	15	48	16
“I thought about the sounds around me.”	14	23	18	36	10
“I thought about the odors around me.”	43	39	10	7	2
“I thought about my heartbeat.”	43	34	8	12	4
“I thought about my breathing.”	26	21	11	28	14
“ I felt pain.” (R)	54	25	7	12	3
“I placed myself in other peoples' shoes.”	45	32	13	9	1
“I felt motivated to participate.”(V)	3	6	21	52	18
“I have difficulty remembering my thoughts.” (V)	33	39	15	11	1
“I have difficulty remembering my feelings.” (V)	34	42	13	10	2
“I had my eyes closed.” (V)	2	2	5	30	62
“I was able to rate the statements.” (V)	0	1	9	55	35

*An (R) designates items that were reverse coded prior to analysis, (V) indicates those items that were used for validation. Ratings ranged from “Completely disagree” (–) to “Completely agree” (++) on a 5-point rating scale*.

### Participants

We obtained ARSQ data from three different settings (see Table 2 for group-specific demographics). The largest data set (*n* = 1367, 908 females, age range 18–87) was obtained from the Dutch version of the questionnaire as part of the online assessment battery of the Netherlands Sleep Registry (www.sleepregistry.org), through which participants could complete the ARSQ at home. The Netherlands Sleep Registry (NSR) is a database aimed at sampling multiple questionnaires in a large cohort comprising the full range from very light to very sound sleepers. Approximately 77% of our participants were identified to not suffer from insomnia based on their score on the Insomnia Severity Index with a cutoff of 14 (Morin et al., 2011), which is in line with reported insomnia prevalence ratings of 9–27% in the general population (Leblanc et al., 2009). In addition to the home setting, we analyzed ARSQ data obtained from a sample of dizygotic twins and their close relatives (*n* = 68, 43 females, age range 19–52) after an eyes-closed resting-state fMRI experiment and while still lying in the scanner. Finally, we analyzed data from a sample of university students (*n* = 89, 50 females, age range 18–55) that partook in studies of resting-state EEG. The study protocols presented here were approved by the institutional review board of the VU University Medical Centre/Academic Medical Centre, Amsterdam, The Netherlands. All participants provided written informed consent prior to participation in the study.

**Table 2:** **Amsterdam Resting-State Questionnaire participant statistics, based on validated data set (*n* = 813)**.

**Group**	**Sample size**		**Age**
	**Total**	**Females**	**Mean (±SD)**
**NETHERLANDS SLEEP REGISTRY**			
Amsterdam Resting-State Questionnaire (ARSQ)	813	622	52.9 (14.2)
Pittsburg Sleep Quality Index (PSQI)	458^*^	352	53.7 (14.3)
Insomnia Severity Index (ISI)	458^*^	352	53.7 (14.3)
Hospital Anxiety and Depression Scale (HADS)	700^*^	523	53.0 (14.1)
Center for Epidemiological Studies Depression Scale (CES-D)	700^*^	523	53.0 (14.1)
Inventory of Depressive Symptomatology (IDS)	699^*^	523	53.3 (14.0)
Temporal Experience of Pleasure Scale (TEPS)	337^*^	258	54.9 (13.8)
RAND-36	699^*^	520	53.5 (13.9)
**fMRI COHORT**			
Amsterdam Resting-State Questionnaire (ARSQ)	68	43	29.7 (8.8)
**EEG COHORT**			
Amsterdam Resting-State Questionnaire (ARSQ)	89	50	21.7 (4.7)

* *Number of participants who filled out both ARSQ (validated data set) and the respective scale*.

### Assessment of resting-state cognition

Participants in the home setting were asked to enable and test their PC audio equipment (i.e., turn on speakers or put on headphones) and received the following instruction: “For this experiment it is required that you are able to sit relaxed with your eyes closed in a quiet and isolated environment during a period of 5 min.” Subsequently, participants could start the experiment with the additional instruction: “Once the resting session has ended you will be notified by a beep. You can stop the sound by clicking ‘Stop.’ Afterwards you can click on ‘Next’ to proceed to the questions. Should you be interrupted by something or someone during the 5 min rest, you may open your eyes, click on the button ‘Stop’ and, subsequently, on the button ‘Restart’ to restart the resting session.” The task instruction immediately preceding the start of the experiment was “The following part of the test lasts 5 min. Relax, try not to fall asleep, click ‘Next’ and immediately close your eyes to start.” The task instruction immediately after the 5 min of eyes-closed rest was: “The 5 min of rest is over. Now several statements will follow regarding potential feelings and thoughts you may have experienced during the resting period. Please indicate the extent to which you agree with each statement.” In order to identify invalid trials, participants in the NSR sample indicated at the end of the questionnaire whether the eyes-closed rest session was interrupted or not, with the option to give a detailed reason. The participants of the fMRI and EEG experiments received identical task instructions for the resting state, except that they were notified by the experimenter when the 5 min had passed. The fMRI participants used a button-box to select their responses in an E-Prime 2.0 (Psychology Software Tools, Pittsburgh, PA) adapted version of the ARSQ after the resting period and while still in the scanner. The EEG participants were placed in a dimly lit room and a comfortable chair with high-density (128-channel) EEG caps mounted and filled out a computerized version of the ARSQ via mouse input. In all three groups, the ARSQ was administered immediately following the 5 min eyes-closed rest.

### Questionnaire data preparation

Data were obtained from 1367 respondents and subsequently conservatively screened based on the validation items of the ARSQ to limit the potentially negative impact on rating accuracy introduced by inability to recall resting-state cognition or reluctance to properly conform to the task-instructions. This was done by (1) filtering out those who reported being interrupted during the trial (*n* = 159), (2) those that did not feel motivated to participate in the study (*n* = 112), (3) participants that had difficulty remembering their thoughts (*n* = 170) or feelings (*n* = 154), (4) removing participants that indicated not being able to rate the statements (*n* = 135), (5) removing participants scoring below “Agree” on the statements “I had my eyes closed” (*n* = 118) and, finally, (6) removing participants scoring consistently at the extremes of the scale (*n* = 2). The NSR participants were encouraged to fill out additional psychometric scales, which resulted in sample sizes ranging from 337–700 (Table 2). Finally, the item “I had my thoughts under control” was reverse coded prior to confirmatory factor analysis and computation of the mean scores, such that all items correlate positively with a given factor.

### EEG recordings

Participants in the EEG setting were recruited from the student population of the VU University Amsterdam as part of an elective course. Measurements took place in our EEG laboratory. Resting-state recordings were performed using two Electrical Geodesics (EGI, Eugene, OR) EEG systems (GES250 AC and GES300 DC), using 128-EGI HydroCel channel sponge-base EEG-caps. Impedances were kept below 75 kOhm (a suitable threshold for these kinds of EEG-caps). Data were recorded at 1000 Hz sampling rate, with a 400 Hz low-pass (GES250) and 0.01 Hz high-pass hardware filter and with 16 Bit resolution and reference at Cz (vertex).

### EEG analysis

The data were subsequently exported to MATLAB (The Mathworks Inc., Natick, MA), down-sampled to 500 Hz and band-pass filtered between 1 and 45 Hz using finite impulse response (fir) filters. Data cleaning and artifact rejection was done using the automated algorithms in the MATLAB toolboxes FASTER (Nolan et al., 2010) to detect and interpolate bad channels and epochs and ADJUST (Mognon et al., 2011) to remove eye movement and muscle artifacts using independent component analysis. Signals were then re-referenced to common average reference. Analyses and EEG biomarker extraction was performed using EEGLAB (Delorme and Makeig, 2004) and the Neurophysiological Biomarker Toolbox (Hardstone et al., 2012, www.nbtwiki.net). Here, we report on the analysis of 95th percentile theta-oscillation life-times as introduced by Montez et al. (2009). Briefly, this biomarker is obtained for each channel by band-pass filtering the signal between 4 and 7 Hz using a fir filter, extracting the amplitude envelope using the Hilbert transform, introducing a threshold at the median of the amplitude envelope, and identifying the start and end of an oscillation burst from the crossings of the amplitude envelope with that threshold. The life-time of a burst is the time between crossings. Finally, the cumulative probability distribution of oscillation life-times is computed and the 95th percentile extracted as the biomarker representing the tendency of oscillations to stay elevated in amplitude for a sustained period of time in each channel. Correlation between cognitive dimensions and biomarkers were based on the Pearson product-moment correlation coefficient. We did not apply a correction for multiple comparisons, because the number of channels with *p*-values below 0.05 was 13 (Figure 7) and the probability of this many or more false positives is only 1% (cf. binomial distribution, Montez et al., 2009).

## Results

### Seven dimensions of resting-state cognition

Raw ARSQ response data (*n* = 1367) were screened based on the five validation items of the ARSQ (see Materials and Methods), resulting in a reference data set (*n* = 813). In order to make a preliminary assessment whether the data could be partitioned into meaningful clusters, we performed an exploratory factor analysis (EFA) with oblique rotation on the reference data set with 45 ARSQ statements (excluding the five used for validation) extracting seven factors (data not shown), which represented the data well [χ^2^ (696, *N* = 813) = 2687.15, Root Mean Square Error of Approximation (*RMSEA*) = 0.059]. In an additional cross-validation step, we recalculated the EFA based on half the reference data set (*n* = 400) and obtained similar fit indices [χ^2^ (696, *N* = 400) = 1633.89, *RMSEA* = 0.058].

These results suggested that our data were suitable for fitting more advanced models and we proceeded with a confirmatory factor analysis (Digman, 1997; Hoekstra et al., 2011; Tully et al., 2011). A number of items (*n* = 18, Table 3) were excluded from the model, as these could either not be used to form interpretable factors with at least three items (the minimum requirement for indicators in a latent variable model), or did not improve reliability estimates of selected factor items, often due to strong skew in the responses (e.g., “I felt ill”).

**Table 3:** **Descriptive statistics of Amsterdam Resting-State Questionnaire (ARSQ) items and factors**.

	**DoM**	**ToM**	**Self**	**Plan**	**Sleep**	**Comf**	**SomA**
**FACTORIAL CORRELATIONS^1^**							
Discontinuity of Mind (DoM)	*–*						
Theory of Mind (ToM)	0.36	–					
Self	0.58	0.59	−				
Planning (Plan)	0.58	0.70	0.81	–			
Sleepiness (Sleep)	*0.32*	*0.11*	0.24	0.21	–		
Comfort (Comf)	*0.72*	−*0.04*	−0.27	–0.20	-0.25	–	
Somatic Awareness (SomA)	0.35	0.27	0.71	0.48	0.31	–0.30	–
**STANDARDIZED FACTOR LOADINGS (ITEM MEAN ± SD, C_α_)**							
	**DoM (0.78)**	**ToM (0.79)**	**Self (0.62)**	**Plan (0.76)**	**Sleep (0.80)**	**Comf (0.82)**	**SomA (0.53)**
I felt restless	0.77 (2.2 ± 1.2)						
I had busy thoughts	0.84 (2.3 ± 1.2)						
I had my thoughts under control (R)	0.51 (3.4 ± 1.0)						
I had rapidly switching thoughts	0.77 (2.9 ± 1.2)						
I had difficulty holding on to my thoughts	0. 55 (2.5 ± 1.1)						
I thought about others		0.86 (2.8 ± 1.2)					
I thought about people I like		0.80 (2.7 ± 1.3)					
I placed myself in other peoples shoes		0.80 (1.9 ± 1.0)					
I thought about my feelings			0.66 (2.6 ± 1.2)				
I thought about my behavior			0.74 (2.3 ± 1.2)				
I thought about myself			0.50 (3.5 ± 1.1)				
I thought about my work/study				0.68 (2.4 ± 1.3)			
I thought about solving problems				0.71 (2.5 ± 1.2)			
I thought about the past				0.61 (2.1 ± 1.1)			
I thought about the future				0.71 (2.7 ± 1.2)			
I had deep thoughts				0.67 (2.2 ± 1.0)			
I thought about things I need to do				0.61 (2.9 ± 1.3)			
I felt tired					0.93 (2.4 ± 1.2)		
I felt sleepy					0.84 (2.4 ± 1.2)		
I had difficulty staying awake					0.69 (1.8 ± 1.0)		
I felt comfortable						0.83 (3.8 ± 0.9)	
I felt relaxed						0.97 (3.7 ± 1.0)	
I felt happy						0.66 (3.3 ± 0.9)	
I thought about my health							0.87 (2.1 ± 1.2)
I was conscious of my body							0.21 (3.6 ± 1.1)
I thought about my heartbeat							0.49 (2.0 ± 1.1)
I thought about my breathing							0.18 (2.9 ± 1.4)
**ITEMS USED FOR DATA VALIDATION (ITEM MEAN** ± **SD)**							
I felt motivated to participate	4.0 ± 0.6						
I have difficulty remembering my thoughts	1.7 ± 0.7						
I have difficulty remembering my feelings	1.7 ± 0.7						
I had my eyes closed	4.7 ± 0.5						
I was able to rate the statements	4.4 ± 0.5						
**ITEMS EXCLUDED FROM MODEL (ITEM MEAN** ± **SD)**							
I thought in images	2.9 ± 1.3						
I thought in words	3.1 ± 1.2						
I felt ill	1.4 ± 0.7						
I enjoyed the session	3.0 ± 1.1						
I had negative feelings	1.8 ± 1.0						
I felt bored	2.3 ± 1.1						
I felt nothing	2.1 ± 1.0						
I felt the same throughout the session	3.5 ± 1.1						
I had thoughts that I would not readily share with others	1.7 ± 1.0						
I had similar thoughts throughout the session	3.3 ± 1.1						
I thought about pleasant things	3.1 ± 1.0						
I thought about the aim of the experiment	2.5 ± 1.3						
I had superficial thoughts	3.0 ± 1.1						
I thought about the present	3.5 ± 1.1						
I thought about nothing	2.0 ± 1.1						
I thought about the sounds around me	3.0 ± 1.2						
I thought about the odors around me	1.8 ± 0.9						
I felt pain	1.8 ± 1.1						

1 *Factor labels abbreviated; R, item response reverse coded prior to analysis, underlined numbers indicate non-significant results*.

**C*_α_, Cronbach's α designates reliability of factor items*.

**Figure 1:**
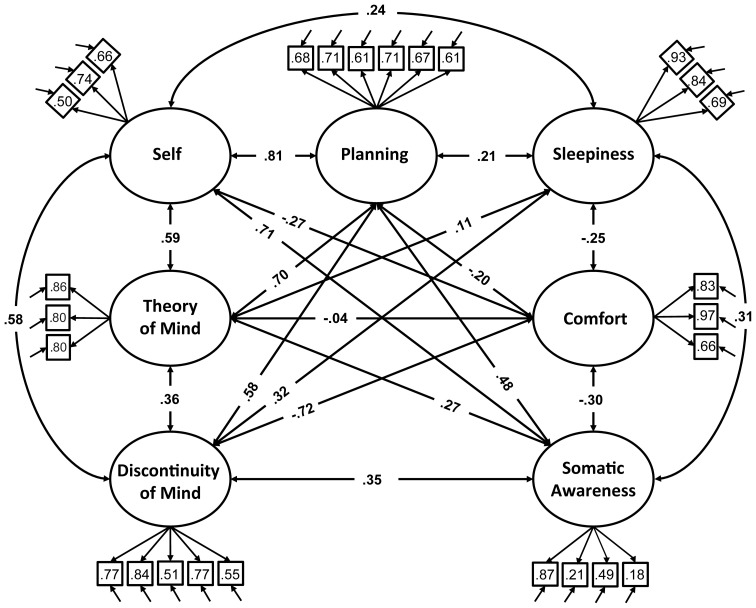
**Path diagram of the specified resting-state cognition model tested in CFA.** Paths between factors (ovals) represent factorial correlations. Numbers within squares indicate the standardized loadings of the particular item on its factor and residuals are depicted by the short single-headed arrows pointing towards items.

**Figure 2:**
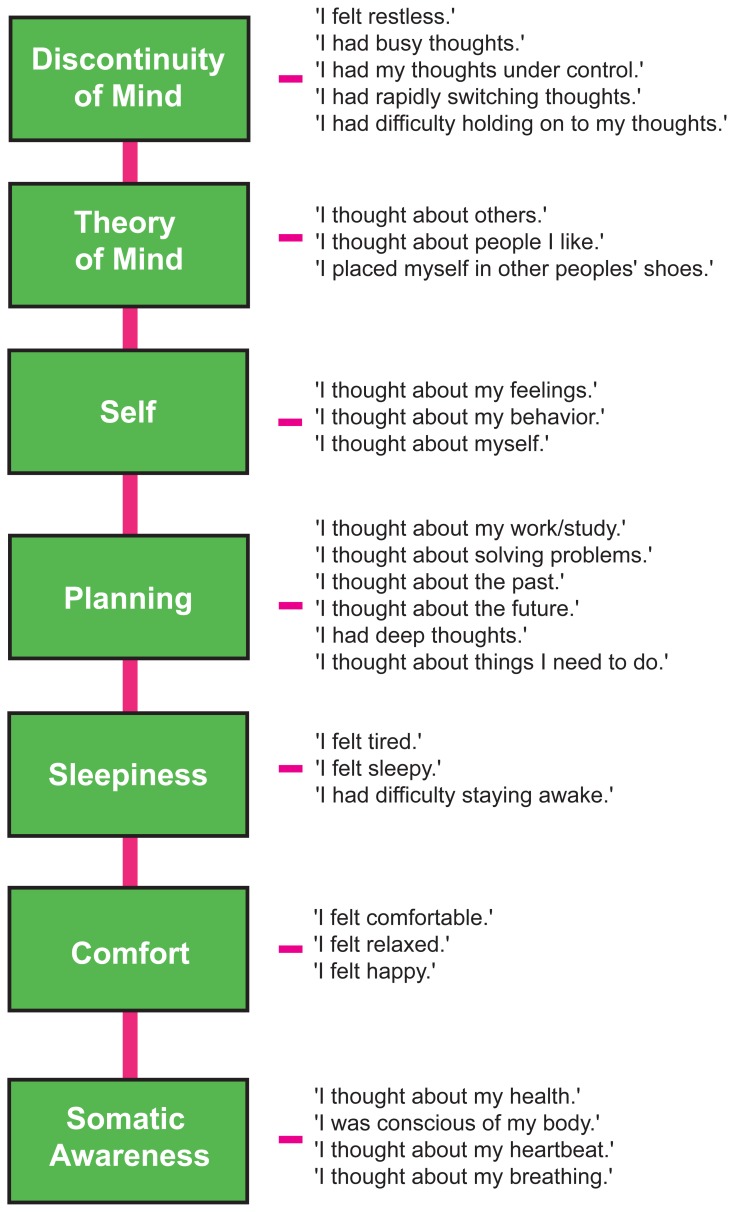
**The Amsterdam Resting-State Questionnaire reveals seven dimensions of resting-state cognition.** The factor analysis of the variance-covariance structure of the data from the self-report ARSQ (*n* = 813) resulted in seven factors, which we labeled as shown in the green boxes. A total of 27 items were included in the final model and the individual items belonging to each factor are listed to the right (the CFA path diagram is shown in Figure 1).

We therefore restricted our selection of items for the model to 27 out of the 45 ARSQ items (Table 3) and created seven factors of resting-state cognition. The resulting model (Figure 1, unit variance identification, zero mean for the latent variables and unit loading identification) fit the data adequately: χ^2^(303, *N* = 813) = 2455.63, *RMSEA* = 0.093, Comparative Fit Index (*CFI*) = 0.88. In line with previous studies, we accepted *RMSEA* fit statistics below 0.1 (Steiger, 1989; Hutchinson and Olmos, 1998) but relaxed the common requirement of having a *CFI* > 0.95 (Schreiber et al., 2006) to *CFI* > 0.85, because the *CFI* is sensitive to correlations among indicators (items) and both the categorical nature and skewness in some items may have contributed to lower observed correlations, attenuating the *CFI*. Based on the content of the items used in the model, we labeled the seven factors: Discontinuity of Mind, Theory of Mind, Self, Planning, Sleepiness, Comfort, and Somatic Awareness (Figure 2). Reliability (Cronbach's α) of the selected items proved well-above 0.75 for most factors (see Table 3), except for “Self” and “Somatic Awareness,” where correlation among the items was lower. However, the assumed construct underlying as reflected by the statements led us to expect that these factors were nevertheless important to retain, i.e., “I thought about myself,” “I thought about my feelings” and “I thought my behavior” may all be sensibly grouped under the factor “Self.”

We further tested the robustness of the model by (1) re-computing the fit statistics for half the reference set (*n* = 400, different cases from EFA validation step) and (2) randomly drawing a subset (*n* = 400) from the reference set for a large number of iterations (*k* = 1000) to estimate the range of fit statistics that can be observed. The results are summarized in Table 4 and show that the split-half sample and the random samples on average fall within these limits, suggesting that the reported model fit is robust within our data set.

**Table 4:** **Averaged fit statistics of (*k* = 1000) randomly selected sets of cases (*n* = 400) for 7-factor resting-state cognition model compared to a split-half validation sample not used for EFA (*n* = 400)**.

**Sample**	**Chi-Square test Mean ± SD [min max]**	**RMSEA Mean ± SD [min max]**	**RMSEA 90% C.I. [min max]**	**CFI Mean ± SD [min max]**
Random	1306.90 ± 82.73	0.091 ± 0.004	0.86 ± 0.004 0.96 ± 0.004	0.88 ± 0.01
	[1045.25 1561.26]	[0.078 0.10]	[0.073 0.11]	[0.84 0.91]
Split-half	1320.06	0.092	[0.087 0.097]	0.86

*RMSEA, Root Mean Square Error of Approximation; CFI, comparative fit index. Degrees of freedom = 303*.

In addition, *inclusion* of the sample of cases previously removed during the screening stage based on the validation items of the ARSQ, left the model fit unaffected [χ^2^(303, *N* = 1112) = 3360.13, *RMSEA* = 0.095, *CFI* = 0.88] suggesting that there was no strong bias from the exclusion of this sample. Similarly, to ensure that the inclusion of participants with a potential sleep disorder did not bias the model of resting-state cognition, we recomputed the CFA excluding all participants scoring in the moderate to clinical range (*n* = 107) of the Insomnia Severity Index (ISI < 15) and observed that the model was still valid [χ^2^(303, *N* = 351) = 1179.01, *RMSEA* = 0.091, *CFI* = 0.87]. Finally, even though a detailed analysis of the particular items that were involved with low rating ability was not possible, we nevertheless tested (independent *t*-test, Bonferroni correction) for mean differences in ARSQ responses between the reference set (*n* = 813) and the set of cases removed for low rating ability (*n* = 135). These results suggest that the excluded subsample experienced significantly lower comfort and Somatic Awareness, but increased Discontinuity of Mind and Sleepiness (Table 5). Interestingly, at the single-item level the top-5 most significant differences were observed for the five validation items. This suggests that some participants were unable to rate the statements because of having lost their memory of thoughts or feelings, or lacking motivation to participate.

**Table 5:** **Participants excluded for indicating inability to rate the ARSQ primarily differ on items that are used for validation (V) and those related to Discontinuity of Mind, Sleepiness and Somatic Awareness**.

	**Reference sample^1^ Mean ± SD**	**Excluded sample^2^ Mean ± SD**	***T*-statistic (df = 946)**
ITEMS^*^			
I was able to rate the statements (V)	3.4 ± 0.5	2.8 ± 0.5	35.16
I have difficulty remembering my thoughts (V)	1.7 ± 0.7	2.8 ± 1.2	−13.89
I have difficulty remembering my feelings (V)	1.7 ± 0.7	2.6 ± 1.2	−12.81
I had my eyes closed (V)	4.7 ± 0.5	4.1 ± 1.1	11.37
I felt motivated to participate (V)	4.0 ± 0.6	3.5 ± 0.9	7.66
I felt happy	3.3 ± 0.9	2.6 ± 1.0	7.31
I felt comfortable	3.8 ± 0.9	3.2 ± 1.1	6.74
I had difficulty holding on to my thoughts	2.5 ± 1.1	3.1 ± 1.1	−6.56
I had my thoughts under control	3.4 ± 1.0	2.8 ± 1.1	6.26
I felt restless	2.2 ± 1.2	2.9 ± 1.2	−6.04
I felt bored	2.3 ± 1.1	2.9 ± 1.2	−5.95
I felt relaxed	3.7 ± 1.0	3.1 ± 1.1	5.81
I enjoyed the session	3.0 ± 1.1	2.4 ± 1.1	5.57
I thought about pleasant things	3.1 ± 1.0	2.6 ± 1.0	5.48
I had negative feelings	1.8 ± 1.0	2.3 ± 1.1	−5.36
I felt tired	2.4 ± 1.2	3.0 ± 1.3	−5.08
I was conscious of my body	3.6 ± 1.1	3.2 ± 1.2	4.47
I felt ill	1.4 ± 0.7	1.7 ± 1.0	−4.31
I felt the same throughout the session	3.5 ± 1.1	3.1 ± 1.2	4.26
I had busy thoughts	2.3 ± 1.2	2.8 ± 1.0	−3.94
I felt sleepy	2.4 ± 1.2	2.8 ± 1.3	−3.87
FACTORS^**^			
Somatic awareness	3.6 ± 0.8	3.0 ± 0.9	7.7
Discontinuity of mind	2.5 ± 0.8	3.0 ± 0.9	–6.98
Sleepiness	2.2 ± 1.0	2.6 ± 1.1	–4.64

1 *Sample with cases removed based on response on Validation items (n = 813)*,

2 Sample of cases removed based on scoring < 3.0 on “I was able to rate the statements” (n = 135). Bonferroni adjustments:

* *α_adjusted_ = 0.001*,

** *α_adjusted_ = 0.007. Only significant mean differences shown*.

In order to simplify future analyses based on the 7-factor model we calculated the mean scores from the ARSQ responses, by averaging the item scores within a factor. These mean scores were strongly correlated with the estimated model factor scores (Table 6) and, therefore, we deemed it acceptable to adopted the more convenient mean scores for all further analyses.

**Table 6:** **Descriptive statistics of correlations between mean scores and estimated model factor scores**.

**Factor**	***r***	***CI***_**95%**_	***t***_**(811)**_	***p***
Discontinuity of mind	0.95	[0.94 0.95]	82.3	<0.001
Theory of mind	0.98	[0.98 0.99]	155.9	<0.001
Self	0.88	[0.86 0.89]	52.5	<0.001
Planning	0.96	[0.95 0.96]	92.8	<0.001
Sleepiness	0.96	[0.96 0.97]	104.8	<0.001
Comfort	0.94	[0.94 0.95]	84.0	<0.001
Somatic awareness	0.66	[0.62 0.69]	24.8	<0.001

*Pearson correlation between estimated factor score derived from CFA model and mean scores, 95th percentile confidence interval of correlation, Student's t-statistic assessing the significance of the correlation (degrees of freedom). Estimated model factor scores are normally distributed and continuous, whereas mean scores are formed by adding responses of items within a factor and dividing by their number, thus yielding discrete responses on the original item scale*.

### Stability and variability of resting-state cognition

The NSR group of participants provided insight into resting-state cognition within a natural and familiar environment. An important motivation behind the development of the ARSQ, however, was to create an effective tool to be used in combination with neuroimaging. Therefore, we also gathered ARSQ data in studies measuring resting-state fMRI (*n* = 68) and EEG (*n* = 89).

**Figure 3:**
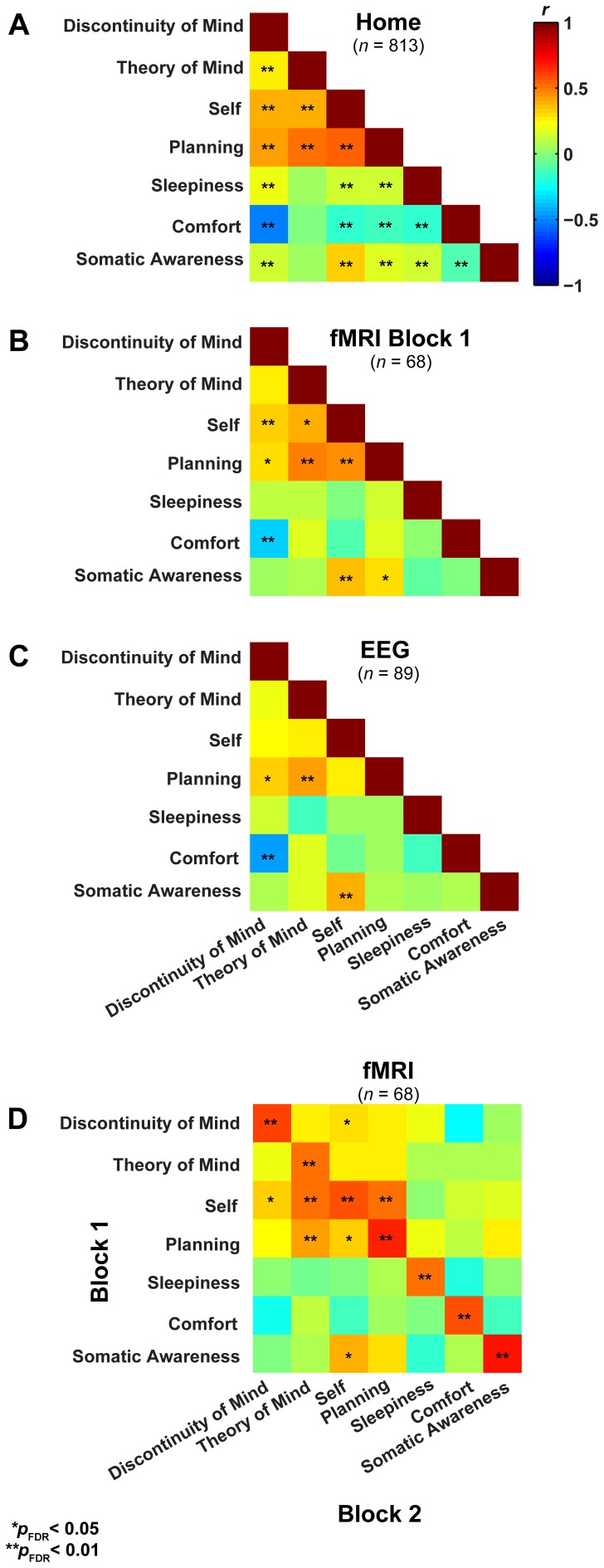
**Resting-state cognition has a similar structure in different experimental settings and shows high test-retest correlation. (A)** Lower triangular Pearson correlation matrix of resting-state cognition factors derived from the NSR participants in their home environment. **(B)** The same correlation matrix derived for subjects filling in the ARSQ immediately after a resting-state fMRI experiment (1st block) and while still lying in the scanner. **(C)** Data from participants in EEG studies reveal a similar pattern of inter-factor correlations to that in **(A)** and **(B)**. **(D)** The correlation of the factor scores between the first and second resting-state block of the fMRI sample shows strong retest correlations (diagonal).

**Figure 4:**
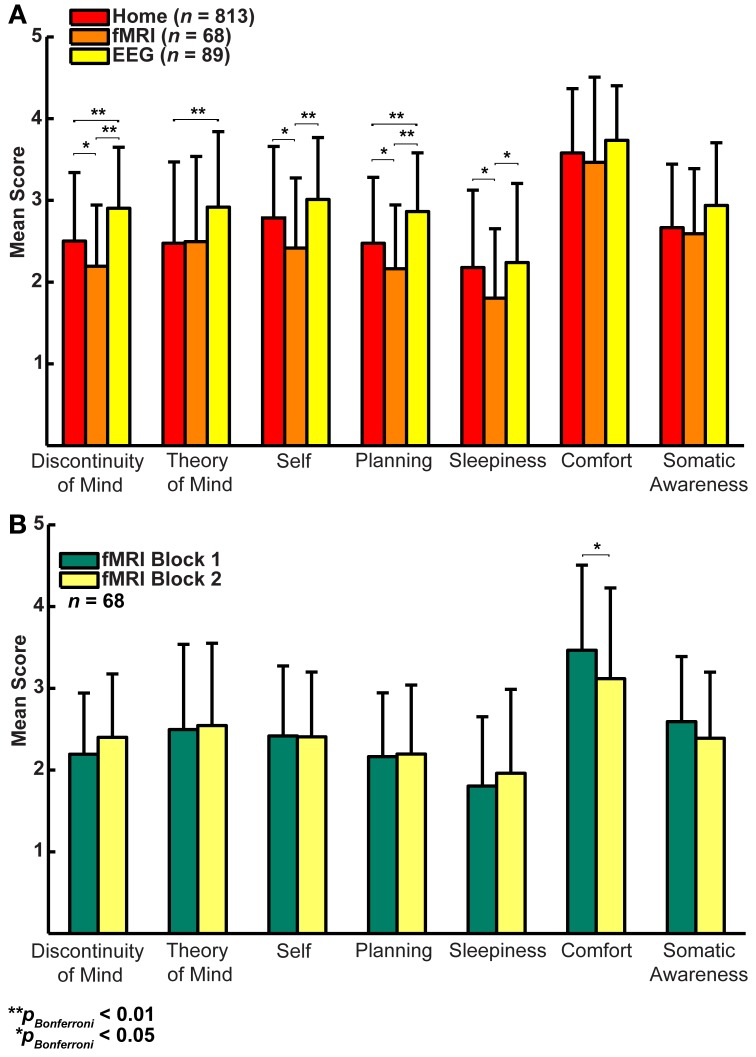
**(A)** Factor score averages (± SD) show that there may be substantial variability in resting-state cognition across the three groups (Home, fMRI, and EEG, *t*-test corrected for family relationships). **(B)** Only Comfort decreases significantly over two resting-state fMRI blocks separated by 45 min (*t*-test with family dependency and Bonferroni correction, ±*SD*).

In a first step, we formally assessed (Lubke and Muthén, 2005) the relationships among the factors between the three groups (Figures 3A–C), which showed that the correlation structure was equivalent across groups [χ^2^_(42)_ = 46.49, *p* = 0.30], but this was not the case for the covariance structure [χ^2^_(56)_ = 74.87, *p* = 0.05]. As the main difference appeared to stem from the factor Comfort, we freed this parameter in a subsequent retesting, which indeed resulted in equal covariance structure across the groups [χ^2^_(55)_ = 60.17, *p* = 0.29]. Next, we tested for mean differences (one-way ANOVA, Bonferroni corrected) between the groups on the mean scores of the seven dimensions and found significant effects for five on them: Discontinuity of Mind: χ^2^_(2)_ = 34.63, *p* < 0.01, Theory of Mind: χ^2^_(2)_ = 16.49, *p* < 0.01, Self χ^2^_(2)_ = 19.17, *p* < 0.01, Planning: χ^2^_(2)_ = 36.0, *p* < 0.01 and Sleepiness: χ^2^_(2)_ = 10.96, *p* < 0.05 (see Figure 4A).

**Figure 5:**
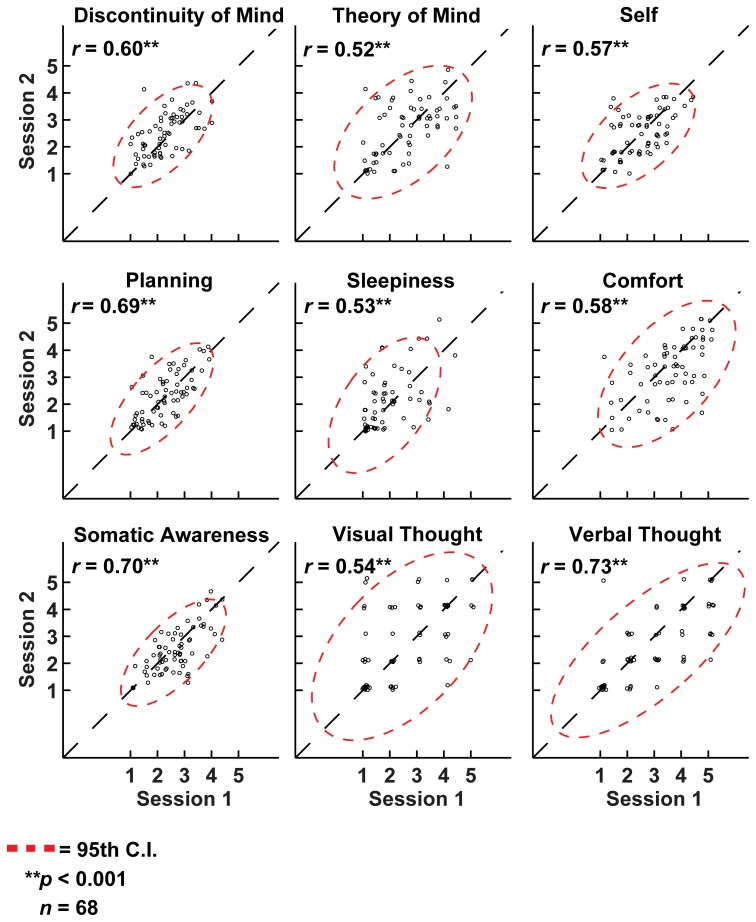
**Cognition over two resting-state sessions was strongly and positively correlated, yet displayed substantial between-subject variation.** As such, variation in resting-state cognition is primarily due to inter-individual differences. Note: to aid visualization, a minute amount of white noise (*M* = 0.1 ± 0.05 SD) was added to the data points.

It may be argued that the minimal instructions involved with assuming a resting state may be so unconstrained as to lead to highly variable and ultimately unpredictable responses on measures of resting state cognition. To address this issue of variability we calculated retest correlations of the ARSQ factors in the fMRI group through a second resting-state examination 45 min after the first block (participants engaged in various cognitive tasks in between). All seven dimensions exhibited remarkably high retest correlations across resting-state blocks (Figure 3D, diagonal and Figure 5) in the range of 0.52–0.70 and corresponding effect sizes (Cohen, 1992) ranging from a medium 27% (e.g., Comfort) to a high level 49% of explained variance (e.g., Somatic Awareness). Although not factors *per se*, the individual items “I thought in words” and “I thought in images” reveal interesting re-test patterns as well, with a very high correlation (*r* = 0.73, *p* < 0.001) for the former item. In addition, the first resting-state session was not statistically different from the second session in terms of correlation [χ^2^_(21)_ = 22.23, *p* = 0.39] or covariance [χ^2^_(28)_ = 33.59, *p* = 0.22] structure. Over the two resting-state sessions, only Comfort showed a significant decrease in mean factor score (Figure 4B).

To further explore the sources of variability we computed the sums of squares from a repeated measures ANOVA for all seven factors in order to assess the amount of variance between and within subjects over the two resting-state blocks (Table 7). Most of the variability over the two resting-state blocks stems from differences between individuals, whereas the remainder is almost completely attributable to within-subject variability—the variance contribution of the resting-state blocks is limited. However, participants engaged in several cognitive tasks in between resting-state blocks, demanding a cognitive switch from mind wandering to task engagement. It is therefore interesting that participants largely returned to the previous mode of resting-state processing, suggesting a trait-like quality of resting-state cognition. These results indicate that besides the stability in structure, resting-state cognition within individuals appears to reflect stable subject characteristics associated with traits or health status.

**Table 7:** **Variance decomposition of resting-state cognition in fMRI group (*n* = 68)**.

	**Sum of squares (%)**			
**Factor**	**Between individuals**	**Resting-state block (within)**	**Residual (within)**	**Total**
Discontinuity of mind	62.1 (78.4)	1.4 (1.8)	15.7 (19.8)	79.3 (100)
Theory of mind	106.8 (75.7)	0.1 (0.1)	34.0 (24.2)	140.9 (100)
Self	71.3 (78.2)	0.00 (0)	19.9 (21.8)	91.2 (100)
Planning	74.5 (84.2)	0.03 (0.0)	14.0 (15.8)	88.5 (100)
Sleepiness	90.4 (75.5)	0.8 (0.7)	28.5 (23.8)	119.7 (100)
Somatic awareness	73.0 (83.4)	1.4 (1.6)	13.1 (15.0)	87.6 (100)
Comfort	122.5 (76.8)	4.1 (2.6)	32.9 (20.6)	159.6 (100)

*Most of the variance in the scores over the two resting-state blocks is due to between-subject variability. Repeated-measures ANOVA sum of squares contribution of inter-individual and intra-individual variability for each resting-state cognition factor mean score. Variance is divided into between-subjects variability and within-subject variability, with the latter subdivided into the effect of the resting-state block and residual variability. Percentages are fractions of total sum of squares*.

### ARSQ correlates with established psychometric scales

**Figure 6:**
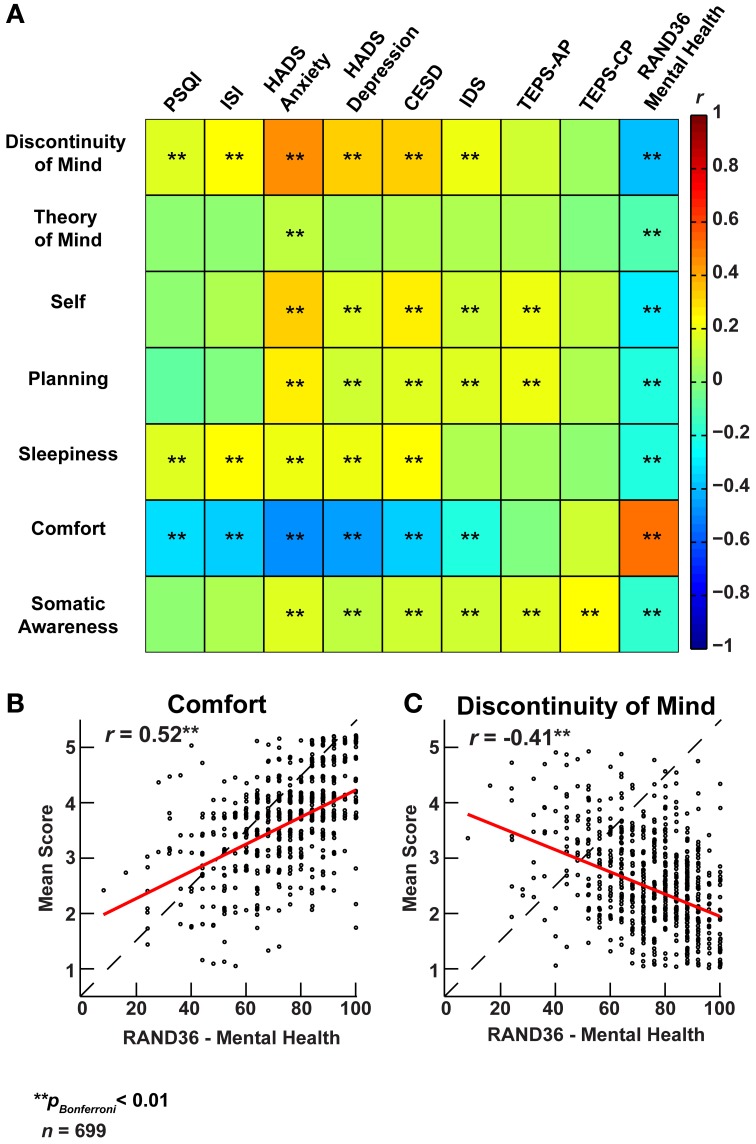
**Factors of resting-state cognition strongly correlate with classical measures of general mental well-being. (A)** Correlation heat map between the seven ARSQ-derived factors of resting-state cognition and nine established psychometric scales of mental well-being (Pearson correlation coefficients). Note the strong and opposite correlations between the factors Discontinuity of Mind and Comfort and the classical scales. **(B)** The correlation pattern of Comfort and **(C)** Discontinuity of Mind reverses for the RAND36 subscale of mental well-being, where high scores should be interpreted as a good mental health. Abbreviations: PSQI, Pittsburgh Sleep Quality Index (Buysse et al., 1989); ISI, Insomnia Severity Index (Bastien et al., 2001); HADS, Hospital Anxiety and Depression Scale (Spinhoven et al., 1997); CESD, Center for Epidemiological Studies Depression Scale (Radloff, 1977); IDS, Inventory of Depressive Symptomatology (Rush et al., 1986); TEPS, Temporal Experience of Pleasure Scale-Anticipatory (AP)/Consummatory Pleasure (CP) (Gard et al., 2006); RAND36, Research And Development 36 (Van Der Zee et al., 1996).

To explore the association of resting-state cognition with mental health, we correlated the ARSQ-derived scores with scores on established psychometric scales (Figure 6A). We observed significant correlations between ARSQ factors and self-report scores of anxiety (Hospital Anxiety and Depression Scale, subscale Anxiety) (Spinhoven et al., 1997), depression (HADS subscale depression, Center for Epidemiological Studies Depression Scale, and Inventory of Depressive Symptomatology) (Radloff, 1977; Rush et al., 1986) and sleep-wake variables (Pittsburgh Sleep Quality Index, and Insomnia Severity Index) (Buysse et al., 1989; Bastien et al., 2001). In particular, Discontinuity of Mind and Comfort exhibited strong associations with indicators of mental health problems. These relationships were further validated by the opposite pattern that emerged when correlated to the Research and Development-36 (Van Der Zee et al., 1996) subscale measuring mental health: high scores of mental wellbeing were accompanied by high scores on Comfort and low scores on Discontinuity of Mind (Figures 6B,C). Interestingly, we note that the TEPS (temporal experience of pleasure scale) (Gard et al., 2006) subscale of “anticipatory pleasure” only correlated with Self and Planning, which are the cognitive components one would expect to be primarily involved in the anticipation of pleasant stimuli.

### Sleepiness correlates with clinically relevant EEG biomarkers

**Figure 7:**
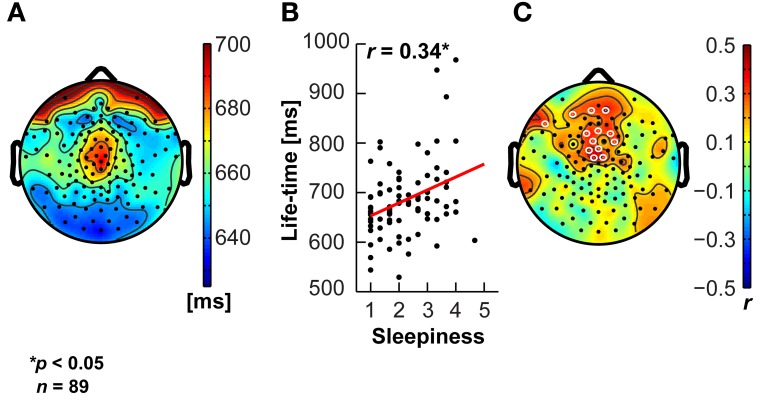
**Sleepiness correlates positively with sustained theta activity.** The theta oscillation life-time biomarker shows a central topography **(A)**, and correlates positively (significant electrodes in white open circles) with the Sleepiness score of the ARSQ in medial fronto-central scalp regions **(B,C)**.

A commonly noted phenomenon in clinical neurophysiology is the slowing of resting-state electroencephalographic or magneto-encephalographic activity in neurological disease (Grunwald et al., 2002; Stoffers et al., 2007; Montez et al., 2009). It is also well-known that states of drowsiness are related to increased activity in lower frequency bands, e.g., in the 4–7 Hz theta range (Strijkstra et al., 2003). We therefore tested the relationship between the ARSQ factor of Sleepiness and theta-band activity (Figures 7A–C, see Materials and Methods) and observed a prominent positive correlation was observed for the 95th percentile theta-burst life-time (Montez et al., 2009). These findings suggest that the Sleepiness score may be used to discover potential confounders or serve as a covariate in those cases where low levels of vigilance may potentially interfere with the interpretation of the results. In addition, these results seem to suggest that even normal individuals may exhibit patterns of brain activity similar to those of patients with neurological impairments (e.g., Alzheimer's disease), simply due to the mediating role of lowered arousal. A detailed description of associations between the seven ARSQ dimensions of resting-state cognition and neuroimaging biomarkers from EEG and fMRI will be provided in future publications.

## Discussion

In the present study, we investigated resting-state cognition experienced during 5 min of eyes-closed rest by means of the ARSQ. Using exploratory and confirmatory factor analysis, we derived seven dimensions: Discontinuity of Mind, Theory of Mind, Self, Planning, Sleepiness, Comfort, and Somatic Awareness. All dimensions showed large inter-individual variation, high retest correlation, and highly similar interdependencies across three experimental settings. Importantly, resting-state cognition was shown to correlate with mental health and EEG biomarkers. Together, these findings suggest that the function of resting-state brain activity in both fundamental and clinical settings can be further elucidated by taking into account resting-state cognition, which is quickly, reliably and informatively quantified by the ARSQ.

Several of the relationships among the ARSQ factors are consistent with previous research. The observed positive correlation between Sleepiness and Discontinuity of Mind is in line with the loss of coherent thought as one enters a state of drowsiness (Yang et al., 2010). Further, the strong anti-correlation of Comfort and Discontinuity of Mind is interesting, because the items belonging to these factors are qualitatively very different. Discontinuity of Mind is mostly concerned with a subjective sense of control over one's thoughts, whereas Comfort clearly focuses on physical and mental well-being. The present data cannot rule out the possibility that the perception of Discontinuity of Mind is also experienced as uncomfortable; however, we speculate that feeling less comfortable may interfere with the flow of thoughts and cause the perception of discontinuity. Furthermore, when subjects had their resting-state cognition sampled the second time—having spent 45 min in the MRI scanner—their Comfort decreased while Discontinuity of Mind showed an increase (*p* = 0.02, uncorrected). In other words, if there is distracting bodily discomfort, the continuity of thought may be perceived to be low.

In the absence of goal-directed behavior, most cognition during rest will qualify as mind wandering. Interestingly, the propensity to mind wander has recently been associated with an elevated experience of unhappiness (Killingsworth and Gilbert, 2010). In our data, however, we note the high scores of Comfort in all three environments (Figure 4), suggesting that it is not the process of mind wandering *per se* that is associated with negative feelings. The negative association of Comfort with sleep complaints as measured by the PSQI and ISI is consistent with previous reports on a diminished capability to recognize comfort in insomnia, which may be related to reduced gray matter volume in the orbitofrontal cortical area that is critical to hedonic evaluation (Kringelbach, 2005; Raymann and Van Someren, 2008; Altena et al., 2010; Stoffers et al., 2012).

The pattern of correlations between the seven dimensions of resting-state cognition assessed by the ARSQ was very similar between the home, EEG, and fMRI settings. In particular, the correlation pattern of Discontinuity of Mind, Theory of Mind, Self, and Planning stayed remarkably stable across different experimental settings. This suggests that mind wandering—a common phenomenon during no or low-demanding externally imposed tasks (Mason et al., 2007; Schooler et al., 2011)—may be rooted in a closely integrated functional system in the brain, which nevertheless shows large inter-individual variation. It has been debated whether the resting state is a well-defined task (Morcom and Fletcher, 2007) or, as some researchers have suggested, a “no task” condition (Stam et al., 2006). Our data strongly suggest that people do indeed perform a multitude of cognitive tasks during the resting state, but that resting-state cognition may exhibit stable, trait-like characteristics, as reflected in the high retest correlation between subsequent fMRI resting-state scans (Figure 5). We speculate that resting-state cognition is analogous to spectral biomarkers of resting-state EEG oscillations, which on the one hand are highly heritable traits (Linkenkaer-Hansen et al., 2007) and, nevertheless, highly sensitive state-changes, such as the progression from wakefulness to sleep. More studies are needed to disentangle state and trait-like properties of resting-state cognition, e.g., by testing the reliability over longer time spans or slight changes in resting-state task instructions.

The significant associations between many dimensions of resting-state cognition and mental-health related scores obtained with well-established psychometric instruments raise interesting perspectives for clinical research. It allows for research into differences in resting-state cognition between patients and healthy controls and whether these can in turn be related to neuroimaging biomarkers, which are often based on resting-state measurements (Greicius, 2008). In this regard, we note the cost efficiency of recording and analyzing ARSQ scores in clinical cohorts, which could pave the way for hypothesis-driven resting-state neuroimaging experiments.

Although research into cognitive constructs and mind perception has yielded fascinating insights (Gray et al., 2007), psychometric instruments aimed at specifically exploring resting-state cognition in neuroimaging are virtually non-existent. To our knowledge, the Resting-State Questionnaire (ReSQ) developed by Delamillieure et al. (2010) is the only other instrument that has been constructed thus far (Delamillieure et al., 2010). The ReSQ and the ARSQ, however, differ substantially from each other. The ReSQ is a semi-structured, supervised and decision-tree based questionnaire focused on visual imagery and inner language/speech–but it should be noted that the ARSQ contains items that assess these specific qualities as well (Figure 5). We considered it important to sample multiple dimensions of resting-state cognition in a standardized self-report fashion and self-assessments have the advantage of reducing the risk of deteriorating the recollection of resting-state cognition by interference of the experimenter or by delayed onset of sampling (Jonides et al., 2008). Finally, the short completion time of the *full* ARSQ (on average less than 4 min) adds much to its usefulness in expensive and time-constrained neuroimaging settings.

To conclude, the ARSQ allows for efficient, standardized assessment of resting-state cognition during the classical eyes-closed resting-state experiment and offers insights into the content of cognition in various contexts and holding predictive value with regard to established clinical instruments. We conjecture that in health and disease, quantification of resting-state cognition will prove invaluable for addressing possible confounds and will aid optimal interpretation of physiological data acquired with resting-state neuroimaging.

### Conflict of interest statement

The authors declare that the research was conducted in the absence of any commercial or financial relationships that could be construed as a potential conflict of interest.

## References

[B1] Aldao A. Nolen-Hoeksema S. Schweizer S. (2010). Emotion-regulation strategies across psychopathology: a meta-analytic review. Clin. Psychol. Rev. 30, 217–237 10.1016/j.cpr.2009.11.004 20015584

[B2] Altena E. Vrenken H. Van Der Werf Y. D. Van Den Heuvel O. A. Van Someren E. J. W. (2010). Reduced orbitofrontal and parietal gray matter in chronic insomnia: a voxel-based morphometric study. Biol. Psychiatry 67, 182–185 10.1016/j.biopsych.2009.08.003 19782344

[B3] Antrobus J. S. (1968). Information theory and stimulus-independent thought. Br. J. Psychol. 59, 423–430

[B4] Bastien C. H. Vallières A. Morin C. M. (2001). Validation of the insomnia severity index as an outcome measure for insomnia research. Sleep Med. 2, 297–307 10.1016/S1389-9457(00)00065-4 11438246

[B5] Brown K. W. Ryan R. M. (2003). The benefits of being present: mindfulness and its role in psychological well-being. J. Pers. Soc. Psychol. 84, 822 10.1037/0022-3514.84.4.822 12703651

[B6] Buckner R. L. Andrews-Hanna J. R. Schacter D. L. (2008). The brain's default network: anatomy, function, and relevance to disease. Ann. N.Y. Acad. Sci. 1124, 1–38 10.1196/annals.1440.011 18400922

[B7] Buckner R. L. Carroll D. C. (2007). Self-projection and the brain. Trends Cogn. Sci. 11, 49–57 10.1016/j.tics.2006.11.004 17188554

[B8] Buysse D. Reynolds C. Monk T. Berman S. Kupfer D. (1989). Pittsburgh sleep quality index (PSQI). Psychiatry Res. 28, 193–213 10.1016/0165-1781(89)90047-4 2748771

[B9] Christoff K. Gordon A. M. Smallwood J. Smith R. Schooler J. W. (2009). Experience sampling during fMRI reveals default network and executive system contributions to mind wandering. Proc. Natl. Acad. Sci. U.S.A. 106, 8719–8724 10.1073/pnas.0900234106 19433790 PMC2689035

[B10] Cohen J. (1992). A power primer. Psychol. Bull. 112, 155–159 10.1037/0033-2909.112.1.155 19565683

[B11] Costa P. T. McRae R. R. (1985). NEO Personality Inventory Manual. Odessa, FL: Psychological Assessment Resources

[B12] Damoiseaux J. S. Rombouts S. A. Barkhof F. Scheltens P. Stam C. J. Smith S. M. (2006). Consistent resting-state networks across healthy subjects. Proc. Natl. Acad. Sci. U.S.A. 103, 13848–13853 10.1073/pnas.0601417103 16945915 PMC1564249

[B13] Delamillieure P. Doucet G. Mazoyer B. Turbelin M. R. Delcroix N. Mellet E. (2010). The resting state questionnaire: an introspective questionnaire for evaluation of inner experience during the conscious resting state. Brain Res. Bull. 81, 565–573 10.1016/j.brainresbull.2009.11.014 20003916

[B14] Delorme A. Makeig S. (2004). EEGLAB: an open source toolbox for analysis of single-trial EEG dynamics including independent component analysis. J. Neurosci. Methods 134, 9–21 10.1016/j.jneumeth.2003.10.009 15102499

[B15] Digman J. M. (1997). Higher-order factors of the big five. J. Pers. Soc. Psychol. 73, 1246–1256 10.1037/0022-3514.73.6.1246 9418278

[B16] Doucet G. Naveau M. Petit L. Zago L. Crivello F. Jobard G. (2012). Patterns of hemodynamic low-frequency oscillations in the brain are modulated by the nature of free thought during rest. Neuroimage 59, 3194–3200 10.1016/j.neuroimage.2011.11.059 22155378

[B17] Fox M. D. Raichle M. E. (2007). Spontaneous fluctuations in brain activity observed with functional magnetic resonance imaging. Nat. Rev. Neurosci. 8, 700–711 10.1038/nrn2201 17704812

[B18] Gard D. E. Gard M. G. Kring A. M. John O. P. (2006). Anticipatory and consummatory components of the experience of pleasure: a scale development study. J. Res. Pers. 40, 1086–1102 10.1016/j.jrp.2005.11.001

[B19] Gray H. M. Gray K. Wegner D. M. (2007). Dimensions of mind perception. Science 315, 619 10.1126/science.1134475 17272713

[B20] Greicius M. D. (2008). Resting-state functional connectivity in neuropsychiatric disorders. Curr. Opin. Neurol. 21, 424–430 10.1097/WCO.0b013e328306f2c5 18607202

[B21] Greicius M. D. Flores B. H. Menon V. Glover G. H. Solvason H. B. Kenna H. (2007). Resting-state functional connectivity in major depression: abnormally increased contributions from subgenual cingulate cortex and thalamus. Biol. Psychiatry 62, 429–437 10.1016/j.biopsych.2006.09.020 17210143 PMC2001244

[B22] Grunwald M. Busse F. Hensel A. Riedel-Heller S. Kruggel F. Arendt T. (2002). Theta-power differences in patients with mild cognitive impairment under rest condition and during haptic tasks. Alzheimer Dis. Assoc. Disord. 16, 40–48 10.1097/00002093-200201000-00006 11882748

[B23] Hardstone R. Poil S.-S. Schiavone G. Nikulin V. V. Mansvelder H. D. Linkenkaer-Hansen K. (2012). Detrended fluctuation analysis: a scale-free view on neuronal oscillations. Front. Physiol. 3:450 10.3389/fphys.2012.00450 23226132 PMC3510427

[B24] Hasenkamp W. Wilson-Mendenhall C. D. Duncan E. Barsalou L. W. (2012). Mind wandering and attention during focused meditation: a fine-grained temporal analysis of fluctuating cognitive states. Neuroimage 59, 750–760 10.1016/j.neuroimage.2011.07.008 21782031

[B25] He B. J. (2011). Scale-free properties of the functional magnetic resonance imaging signal during rest and task. J. Neurosci. 31, 13786–13795 10.1523/JNEUROSCI.2111-11.2011 21957241 PMC3197021

[B26] Hoekstra R. A. Vinkhuyzen A. A. E. Wheelwright S. Bartels M. Boomsma D. Baron-Cohen S. (2011). The construction and validation of an abridged version of the autism-spectrum quotient (AQ-short). J. Autism Dev. Disord. 41, 589–596 10.1007/s10803-010-1073-0 20697795 PMC3076581

[B27] Hutchinson S. R. Olmos A. (1998). Behavior of descriptive fit indexes in confirmatory factor analysis using ordered categorical data. Struct. Equ. Model. 5, 344–364 10.1080/10705519809540111

[B28] Jonides J. Lewis R. L. Nee D. E. Lustig C. A. Berman M. G. Moore K. S. (2008). The mind and brain of short-term memory. Annu. Rev. Psychol. 59, 193–224 10.1146/annurev.psych.59.103006.093615 17854286 PMC3971378

[B29] Killingsworth M. A. Gilbert D. T. (2010). A wandering mind is an unhappy mind. Science 330, 932 10.1126/science.1192439 21071660

[B30] Klumpers L. E. Cole D. M. Khalili-Mahani N. Soeter R. P. Te Beek E. T. Rombouts S. A. (2012). Manipulating brain connectivity with δ9-tetrahydrocannabinol: a pharmacological resting state FMRI study. Neuroimage 63, 1701–1711 10.1016/j.neuroimage.2012.07.051 22885247

[B31] Kringelbach M. L. (2005). The human orbitofrontal cortex: linking reward to hedonic experience. Nat. Rev. Neurosci. 6, 691–702 10.1038/nrn1747 16136173

[B32] Leblanc M. Mérette C. Savard J. Ivers H. Baillargeon L. Morin C. M. (2009). Incidence and risk factors of insomnia in a population-based sample. Sleep 32, 1027 19725254 10.1093/sleep/32.8.1027 PMC2717193

[B33] Linkenkaer-Hansen K. Monto S. Rytsala H. Suominen K. Isometsa E. Kahkonen S. (2005). Breakdown of long-range temporal correlations in theta oscillations in patients with major depressive disorder. J. Neurosci. 25, 10131–10137 10.1523/JNEUROSCI.3244-05.2005 16267220 PMC6725784

[B34] Linkenkaer-Hansen K. Nikouline V. V. Palva J. M. Ilmoniemi R. J. (2001). Long-range temporal correlations and scaling behavior in human brain oscillations. J. Neurosci. 21, 1370 11160408 10.1523/JNEUROSCI.21-04-01370.2001 PMC6762238

[B32a] Linkenkaer-Hansen K. Smit D. J. Barkil A. Van Beijsterveldt T. E. Brussaard A. B. Boomsma D. I. (2007). Genetic contributions to long-range temporal correlations in ongoing oscillations. J. Neurosci. 27, 13882–13889 10.1523/JNEUROSCI.3083-07.2007 18077700 PMC6673639

[B35] Lubke G. H. Muthén B. O. (2005). Investigating population heterogeneity with factor mixture models. Psychol. Methods 10:21 10.1037/1082-1989X.10.1.21 15810867

[B36] Mason M. F. Norton M. I. Van Horn J. D. Wegner D. M. Grafton S. T. Macrae C. N. (2007). Wandering minds: the default network and stimulus-independent thought. Science 315, 393–395 10.1126/science.1131295 17234951 PMC1821121

[B37] Mognon A. Jovicich J. Bruzzone L. Buiatti M. (2011). ADJUST: an automatic EEG artifact detector based on the joint use of spatial and temporal features. Psychophysiology 48, 229–240 10.1111/j.1469-8986.2010.01061.x 20636297

[B38] Montez T. Poil S. S. Jones B. F. Manshanden I. Verbunt J. P. Van Dijk B. W. (2009). Altered temporal correlations in parietal alpha and prefrontal theta oscillations in early-stage Alzheimer disease. Proc. Natl. Acad. Sci. U.S.A. 106, 1614–1619 10.1073/pnas.0811699106 19164579 PMC2635782

[B39] Mor N. Winquist J. (2002). Self-focused attention and negative affect: a meta-analysis. Psychol. Bull. 128, 638–662 10.1037/0033-2909.128.4.638 12081086

[B40] Morcom A. M. Fletcher P. C. (2007). Does the brain have a baseline? Why we should be resisting a rest. Neuroimage 37, 1073–1082 10.1016/j.neuroimage.2006.09.013 17681817

[B41] Morin C. M. Belleville G. Bélanger L. Ivers H. (2011). The insomnia severity index: psychometric indicators to detect insomnia cases and evaluate treatment response. Sleep 34, 601 21532953 10.1093/sleep/34.5.601 PMC3079939

[B42] Nolan H. Whelan R. Reilly R. B. (2010). FASTER: fully automated statistical thresholding for EEG artifact rejection. J. Neurosci. Methods 192, 152–162 10.1016/j.jneumeth.2010.07.015 20654646

[B43] Ogedegbe G. Pickering T. G. Clemow L. Chaplin W. Spruill T. M. Albanese G. M. (2008). The misdiagnosis of hypertension: the role of patient anxiety. Arch. Intern. Med. 168, 2459–2465 10.1001/archinte.168.22.2459 19064830 PMC4843804

[B44] Palva J. M. Zhigalov A. Hirvonen J. Korhonen O. Linkenkaer-Hansen K. Palva S. (2013). Neuronal long-range temporal correlations and avalanche dynamics are correlated with behavioral scaling laws. Proc. Natl. Acad. Sci. U.S.A. 110, 3585–3590 10.1073/pnas.1216855110 23401536 PMC3587255

[B45] Radloff L. S. (1977). The CES-D scale. Appl. Psychol. Meas. 1, 385–401 10.1177/01466216770010030611454239

[B46] Raichle M. E. Macleod A. M. Snyder A. Z. Powers W. J. Gusnard D. A. Shulman G. L. (2001). A default mode of brain function. Proc. Natl. Acad. Sci. U.S.A. 98, 676–682 10.1073/pnas.98.2.676 11209064 PMC14647

[B47] Raymann R. J. E. M. Van Someren E. J. W. (2008). Diminished capability to recognize the optimal temperature for sleep initiation may contribute to poor sleep in elderly people. Sleep 31, 1301 18788655 PMC2542970

[B48] Rush J. A. Giles D. E. Schlesser M. A. Fulton C. L. Weissenburger J. Burns C. (1986). The inventory for depressive symptomatology (IDS): preliminary findings. Psychiatry Res. 18, 65–87 10.1016/0165-1781(86)90060-0 3737788

[B49] Schooler J. W. Smallwood J. Christoff K. Handy T. C. Reichle E. D. Sayette M. A. (2011). Meta-awareness, perceptual decoupling and the wandering mind. Trends Cogn. Sci. 15, 319–326 21684189 10.1016/j.tics.2011.05.006

[B50] Schreiber J. B. Nora A. Stage F. K. Barlow E. A. King J. (2006). Reporting structural equation modeling and confirmatory factor analysis results: a review. J. Educ. Res. 99, 323–337 10.3200/JOER.99.6.323-338

[B51] Smallwood J. Schooler J. W. (2006). The restless mind. Psychol. Bull. 132, 946 10.1037/0033-2909.132.6.946 17073528

[B52] Smallwood J. Tipper C. Brown K. Baird B. Engen H. Michaels J. R. (2013). Escaping the here and now: evidence for a role of the default mode network in perceptually decoupled thought. Neuroimage 69, 120–125 10.1016/j.neuroimage.2012.12.012 23261640

[B53] Smit D. J. A. Linkenkaer-Hansen K. De Geus E. J. C. (2013). Long-range temporal correlations in resting-state alpha oscillations predict human timing-error dynamics. J. Neurosci. 33, 11212–11220 10.1523/JNEUROSCI.2816-12.2013 23825424 PMC6618606

[B54] Smith S. M. Fox P. T. Miller K. L. Glahn D. C. Fox P. M. Mackay C. E. (2009). Correspondence of the brain's functional architecture during activation and rest. Proc. Natl. Acad. Sci. U.S.A. 106, 13040–13045 10.1073/pnas.0905267106 19620724 PMC2722273

[B55] Spinhoven P. H. Ormel J. Sloekers P. P. A. Kempen G. I. J. M. Speckens A. E. M. Van Hemert A. M. (1997). A validation study of the hospital anxiety and depression scale (HADS) in different groups of Dutch subjects. Psychol. Med. 27, 363–370 10.1017/S0033291796004382 9089829

[B56] Stam C. J. Jones B. F. Manshanden I. Van Cappellen Van Walsum A. M. Montez T. Verbunt J. P. (2006). Magnetoencephalographic evaluation of resting-state functional connectivity in Alzheimer's disease. Neuroimage 32, 1335–1344 10.1016/j.neuroimage.2006.05.033 16815039

[B57] Steiger J. (1989). EzPATH: A Supplementary Module for SYSTAT and SYGRAPH (computer program manual). Evanston, IL: Systat. Inc.

[B58] Stoffers D. Bosboom J. L. Deijen J. B. Wolters E. C. Berendse H. W. Stam C. J. (2007). Slowing of oscillatory brain activity is a stable characteristic of Parkinson's disease without dementia. Brain 130, 1847–1860 10.1093/brain/awm034 17412733

[B59] Stoffers D. Moens S. Benjamins J. Van Tol M. J. Penninx B. W. H. J. Veltman D. J. (2012). Orbitofrontal gray matter relates to early morning awakening: a neural correlate of insomnia complaints? Front. Neurol. 3:105 10.3389/fneur.2012.00105 23060850 PMC3463899

[B60] Strijkstra A. M. Beersma D. G. M. Drayer B. Halbesma N. Daan S. (2003). Subjective sleepiness correlates negatively with global alpha (8-12 Hz) and positively with central frontal theta (4-8 Hz) frequencies in the human resting awake electroencephalogram. Neurosci. Lett. 340, 17–20 10.1016/S0304-3940(03)00033-8 12648748

[B61] Teasdale J. Dritschel B. Taylor M. Proctor L. Lloyd C. Nimmo-Smith I. (1995). Stimulus-independent thought depends on central executive resources. Mem. Cogn. 23, 551–559 10.3758/BF03197257 7476241

[B62] Tully P. J. Winefield H. R. Baker R. A. Turnbull D. A. De Jonge P. (2011). Confirmatory factor analysis of the beck depression inventory-II and the association with cardiac morbidity and mortality after coronary revascularization. J. Health Psychol. 16, 584–595 10.1177/1359105310383604 21346014

[B63] Van Der Zee K. I. Sanderman R. Heyink J. (1996). A comparison of two multidimensional measures of health status: the Nottingham health profile and the RAND 36-item health survey 1.0. Q. Life Res. 5, 165–174 10.1007/BF00435982 8901380

[B64] Yang C.-M. Han H.-Y. Yang M.-H. Su W.-C. Lane T. (2010). What subjective experiences determine the perception of falling asleep during sleep onset period? Conscious. Cogn. 19, 1084–1092 10.1016/j.concog.2009.12.017 20093044

